# Circulating miRNAs as Biomarkers for Endometriosis and Endometriosis-Related Ovarian Cancer—An Overview

**DOI:** 10.3390/jcm8050735

**Published:** 2019-05-23

**Authors:** Marius Alexandru Moga, Andreea Bălan, Oana Gabriela Dimienescu, Victoria Burtea, Roxana Maria Dragomir, Costin Vlad Anastasiu

**Affiliations:** Department of Medical and Surgical Specialties, Faculty of Medicine, Transilvania University of Brasov, 500019 Brasov, Romania; moga.og@gmail.com (M.A.M.); dr.andreeabalan@gmail.com (A.B.); roxana.gidinceanu@unitbv.ro (R.M.D.); canastasiu@gmail.com (C.V.A.)

**Keywords:** endometriosis, endometriosis-related ovarian neoplasm, miRNA, reverse transcription polymerase chain reaction (RT-PCR), biomarker

## Abstract

Early detection and accurate diagnosis are pivotal in the management of endometriosis and endometriosis-related ovarian neoplasms (ERONs), yet there is no clear common ground regarding their pathogenesis. Endometriosis is a debilitating pathology that profoundly impairs the quality of life. Although the spontaneous resolution of endometriosis is possible, studies suggest that it can be a progressive condition, and ERONs can develop. The gold standard for diagnosis remains as the invasive method of laparoscopy followed by histological confirmation. In recent years, novel biomarkers have been discovered. MicroRNAs (miRNA) represent important epigenetic modulators of gene expression and are very attractive as biomarkers due to their lower complexity, tissue specificity, and stability in bodily fluids. Several studies have advanced the possibility of miRNAs becoming potential biomarkers in endometriosis and ERONs. Our aim is to bring these studies together in order to have a better understanding of whether, how, and when miRNAs might be used as biomarkers for these pathologies. Methods: We selected the reviewed papers from Google Academic, PubMed, and CrossRef. A total of eight studies met the inclusion criteria. Results: MiR-200 family, miR-143, 145, miR-20a, and miR199a were the most commonly dysregulated miRNAs in endometriosis, and miR-200 family was found to be dysregulated in both ERONs and endometriosis. Conclusions: No single miRNA was considered as a sole biomarker for this pathology. However, since the prognostic value of biomarkers is generally enhanced if more are assessed at the same time, a panel of miRNAs could be a better indicator of the disease.

## 1. Introduction

Endometriosis is an inflammatory disease that regularly associates chronic pelvic pain, dysmenorrhea, and infertility. This gynecological pathology is characterized by lesions of endometrial-like tissue located in different sites outside of the uterus [1]. The most commonly agreed upon pathogenic theory of endometriosis is based on the phenomenon of retrograde menstruation. The diagnosis is delayed by 8–10 years due to the misinterpretation of endometriosis symptoms as common menstrual cramps [2].

Over the past few decades, a clearer picture of the epidemiology of endometriosis has emerged. Literature review suggests that African and Hispanic [3] women have lower rates of endometriosis, while Asian women have higher rates compared to white women [4]. It is estimated that 52.7% of women are between 18–29 years old at the time of diagnosis [5]. Even though the prevalence of this disease is not accurately expressed, it is believed that approximately 7% to 15% of women of reproductive age [6] have endometriosis with a significantly increased number of infertility cases [7]. 

Although spontaneous resolution of the disease is possible, recent reports suggest that endometriosis can be a progressive condition [8], and endometriosis-related ovarian neoplasms (ERONs) can develop.

Endometriosis, like cancer, is characterized by cell invasion and unrestrained growth. Furthermore, endometriosis and cancer present some similarities, such as the development of new blood vessels and a decreased apoptosis. Despite these similarities, the disease is not considered a malignant disorder, but it can progress to carcinogenesis, and endometriotic lesions may become malignant [9]. Endometriosis has mixed traits of benign disease and malignancy, and its pathogenesis consists of uncontrolled cell proliferation and distant spread of cells. However, it is not capable of producing severe catabolic disturbances, metabolic disorders, or death. Sampson has recognized the possibility of transformation of endometriotic tissue into malignant ovarian cancer for almost 100 years, and endometriosis is considered to possess a premalignant potential [10,11].

Ovarian cancer represents the fifth cause of cancer-related death in women. It also comprises a histologically and genetically broad range of tumors, including those of epithelial, sex cord-stromal, and germ cell origin [12]. In 1925, Sampson first described the ERONs and the diagnosis criteria for carcinomatous development in endometriosis: similar histological pattern, the coexistence of carcinoma and endometriosis into the same place in the ovary, as well as the exclusion of a second malignant tumor elsewhere [13]. In addition to these criteria, the morphological changes of benign endometriosis lesions contiguous with cancerous tissue are a prerequisite for the adjudication of a malignancy originating from endometriosis. This hypothesis was postulated after the Sampson criteria by Scott in 1953 [14]. 

In respect to ERONs incidence, it is surmised that approximately 60% to 80% of these carcinomas occur in the presence of atypical ovarian endometriosis [15], and tumors are predominantly clear cell and endometrioid types [16]. A review that included 29 studies from 1973–2002 on the prevalence of endometriosis in epithelial ovarian cancers revealed a prevalence of 4.5% serous, 1.4% mucinous, 35.9% clear-cell, and 19% endometrioid ovarian carcinomas [17].

Ovarian epithelial tumors are classified into two major categories. Type I ovarian carcinoma contains clear cell carcinoma, endometrioid carcinoma, mucinous carcinoma, and low-grade serous carcinoma. Type II ovarian carcinoma include the most lethal type of ovarian neoplasms, represented by high-grade serous carcinoma [18]. Both type I and type II ovarian tumors are characterized by different precursor lesions and by distinct molecular genetic alterations that account for their unique clinical manifestation and diagnosis pattern. 

A growing body of evidence from both clinicopathological and molecular studies suggests that most ERONs develop from endometriotic cyst epithelium through different stages of tumor progression. Molecular analyses of ERONs have identified several molecular genetic alterations that lead to aberrant activation or inactivation of different pathways [19]. The proliferation of tumor cells and the relationship between these cells and the components of the extracellular matrix represent significant events in the carcinogenesis progress [20]. Thus, understanding the molecular mechanism and the pathogenesis involved in the development of ERON is paramount for the discovery of new biomarkers that might aid early detection and adequate management of the disease.

Recently, new modulators of gene expression—microRNAs (miRNAs)—have been discovered, and their stability and specificity might lend significant potential biomarker value for endometriosis. Furthermore, several studies investigated various circulating miRNAs as biomarkers for ERONs, but the disagreement between these papers upholds the demand for larger and well-controlled clinical trials. 

Early detection and accurate diagnosis are pivotal in the management of endometriosis and ERONs, yet there is no clear common ground regarding their pathogenesis. Despite being frequently encountered pathologies worldwide with serious consequences and high financial burden, the gold standard for diagnosis remains as the invasive method of laparoscopy followed by a histological examination. Hence, researchers have concentrated their efforts in bridging the gap between different possible diagnostic techniques, one of these being the development of noninvasive biomarkers. 

While the literature review offers a large array of studies on the importance of miRNAs in both endometriosis and ERON, there is little consensus regarding the results of these studies. This can be attributed, in part, to the different technologies employed, as well as the heterogeneity of cell types or variations in circadian rhythm. Our aim is to bring these studies together and create a common denominator to have a better understanding of whether, how, and when these biomarkers might be used. The prognostic value of biomarkers is usually increased if more are assessed at the same time. This becomes particularly important in high incidence pathologies with long lag time between the onset of nonspecific symptoms and diagnosis, such as endometriosis and ERON. 

## 2. Material and Methods

This study is a literature review on endometriosis and ERONs, and it was conducted based on previously published articles. With an ongoing interest in the field of pathogenic molecular mechanisms, we aimed at addressing this topic regarding endometriosis and ERON, given the high burden that these pathologies pose on both the patient and the medical system alike.

While literature offers a long array of studies on the diagnosis and the treatment of both of these two diseases, it has proven to be rather scarce in assessing the potential role of miRNAs, ARID1A, and PIK3/ART as biomarkers for endometriosis and ERONs. Our search for related articles in databases such as Google Academic, PubMed, and CrossRef was performed using the Medical Subject Headings (MeSH) keywords, “endometriosis”, “endometriosis-related ovarian neoplasms”, “biomarker”, “miRNA”, and “RT-PCR”. Furthermore, our inclusion criteria consisted of full-text original English written studies based on humans, assessing the dysregulation of serum miRNAs in endometriosis (on both cases and controls) using RT-PCR and microarray for the miRNAs profiling. Only one notable exception was made for one paper that analyzed the level of miRNAs in baboons with induced endometriosis following a 90 days treatment with simvastatin. Despite the research being performed on primates, it supports the idea that miRNAs could be used as diagnostic and prognostic biomarkers for these gynecological pathologies and thus was included in our analysis. A total number of eight studies met all our criteria.

## 3. Molecular Mechanism and Pathogenesis of ERONs Development

Endometriosis is characterized by the presence of ectopic endometrial-like tissue outside of the uterine cavity [21] with the ovary and the pelvic peritoneum being the most common locations. Various other ectopic sites, such as the diaphragm, the pleura, the pericardium, or even the brain, could also be involved [22]. Although the malignant potential of the endometriotic tissue has been recognized, and extensive research has been conducted, the real mechanisms behind it have yet to be uncovered [23].

The most common pathogenic factors of both endometriosis and ovarian cancer include familial predisposition, molecular level genetic alterations, increased angiogenesis, increased cell adhesion, and hormonal factors [24]. 

Over the last few decades, many types of synthetic chemicals with an environmentally harmful potential have been manufactured. Some of these chemicals have a proven ability to disturb the fine balance of the endocrine system, resulting in the disruption of estrogen action and various anomalies of the female reproductive system, fertility, and the development of genital malignancies [25]. The processes that turn endometriosis into a cancer precursor are ongoing topics of intense research. However, despite the advancements in the molecular analysis of ERONs, they remain largely elusive [26]. 

Epithelial ovarian cancer (EOC) remains the first cause of death from gynecological malignancies, with clear cell carcinoma (CCC) and endometrioid ovarian carcinoma (EnOC) being the most prevalent histological types in patients with associated endometriosis [26]. One of the mechanisms of the endometriosis progression to ERONs is based on the increased level of iron-mediated oxidative stress due to repeated bleeding episodes, which modifies the genomic DNA. The damage of DNA or loss of heterozygosity (LOH) in the ectopic foci of the endometrium caused by iron-induced oxidative stress may be a critical factor in the carcinogenic process [27,28]. Furthermore, a subsequent estrogen-receptor (ER) depletion may be observed, and the loss of estrogen function may play a critical role in the progression of carcinogenesis and the aggressiveness of ERONs [29].

Type I ovarian carcinomas tend to be slow-growing and often share mutations in different genes, such as PTEN, ARID1A, KRAS, BRAF, PIK3CA, CTNNB1, PPP2R1A, β-catenin/Wnt, and microsatellite instability. Recent studies have suggested that type II ovarian carcinomas are mainly high-grade neoplasms characterized by aggressive behavior, nearly ubiquitous presence of p53 mutations, and a high level of genetic instability, while type I ovarian carcinomas do not possess a p53 mutation [30,31,32,33]. 

**Figure 1:**
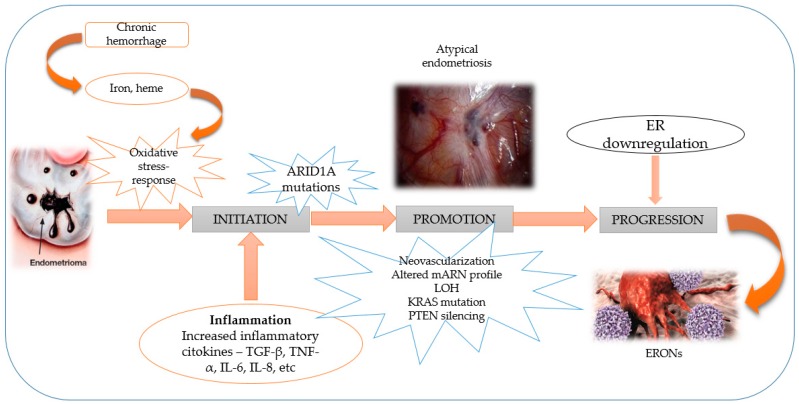
The mechanism of the progression of endometriosis to endometriosis-related ovarian cancer (adapted after Tanase et al. [26] and Samartzis et al. [34]). The process is initiated by heme and iron-mediated oxidative stress, leading to chronic inflammation and repeated hemorrhage. The oxidative stress produces alterations at the DNA level. These free radicals species are generated by an integrated antioxidant defense mechanism, and their production is a prerequisite in modulating different biochemical functions [35]. During the malignant transformation of endometriosis, estrogen receptor (ER) down-regulation is observed. Loss of estrogen function, loss of heterozygosity (LOH), and the mutation of other specific genes represent the trigger factors for the switch from endometriosis to endometriosis-related ovarian neoplasms (ERONs). Additional genes, such as ARID1A, abnormalities, or other molecular alterations may lead to the progression towards ovarian cancer.

Figure 1 is a schematic representation of the mechanism in carcinogenesis of the endometriosis lesions. 

## 4. ARID1A Mutations in ERONs

ARID1A is a protein that protects cells from becoming cancerous. When different mutations occur in the ARID1A gene, the protein cannot be formed and detected by immunohistochemistry. ARID1A mutations are identified in ovarian clear cell carcinoma with a high frequency [36]. The gene consists of 20 exons, and approximately 30% of the clear cell carcinomas of the ovary revealed homozygous mutations of this gene. The ARID1 subfamily includes ARID1A and ARID1B, two subunits of the SWI/SNF chromatin remodeling complex [37,38], and data suggest that ARID1A may act as a tumor suppressor [39]. 

Mutations of ARID1A seem to be frequently involved in ERONs, and almost 73% of heterozygous mutated ovarian tumors are characterized by a loss of protein expression without the loss of heterozygosity [40]. The frequency of loss of ARID1A expression in the endometriosis lesions is an ongoing concern in many studies, as it is suspected that it might be the major genetic alteration that determines the evolution of endometriosis to malignancy [41]. 

Guan et al. sequenced the ARID1A gene in a total of 93 tumor samples with various locations and concluded that none of the 56 ovarian tumors revealed somatic mutations in ARID1A. They correlated the status of ARID1A with the immunohistochemistry and found a significant correlation of ARID1A expression with its mutational status in 51 out of 56 ovarian tumors [42]. Guan et al. also revealed that ARID1A functions as a tumor suppressor. This protein interacts with the p53 protein, and it is capable of suppressing the cellular proliferation through p53-dependent transcriptional regulation of CDKN1A and SMAD3. Thus, the inactivating mutations of ARID1A and TP53 are synonymous, as mutations in TP53 or ADID1A abolish the transcription of tumor suppressors and allow uncontrolled proliferation, resulting in ERONs [43]. 

The results suggest that the loss of ARID1A expression and ARID1A mutations in endometriosis may be involved in the development of ERONs. Furthermore, immunohistochemically, the loss of ARID1A expression may be correlated with truncating ARID1A mutations. 

The link between the loss of ARID1A expression, the development of ERONs, and the circulating miRNAs is not well understood yet. Two miRNAs have been found elevated in endometriosis, miR-221 and miR-222, and their role in increasing cell proliferation could explain the pathophysiology of endometriosis. These circulating miRNAs target ARID1A and down-regulate its expression [44]. Therefore, in women with endometriosis and high levels of miR-221 and miR-222, the loss of ARID1A expression could determine the progression to ERON. Although no study has illustrated upregulation of these miRNAs related to ERONs, further investigation regarding the potential of miR-222 and miR-221 being used as biomarkers for ERONs should be conducted.

## 5. PIK3/AKT-Pathway Mutations in ERONs

Phosphatidylinositol 3-kinase (PI3K)/AKT is a pathway that supports many mechanisms in the process of carcinogenesis [43]. PI3K/AKT pathway mutations have been reported as determining factors of ERONs development. In CCC and EOC, the activation of the PI3K/AKT pathway through mutation of PIK3CA, AKT, and inactivating mutations of PTEN are frequently involved [44]. Studies reported that the mutations in PIK3CA encoding p110α and the activation of the PI3K/AKT pathway by loss of PTEN expression appeared in 33–40% of EOC and 40% of CCC [45,46]. 

There is increasing evidence that ARID1A mutations and PIK3/AKT-pathway mutations may coexist and may be involved in the development of ERONs. Guan et al. [43] tested the possibility of molecular dependency of ARID1A and the PI3K/PTEN pathway in ARID1A knockout mice and ARID1A/PTEN double knockout mice models. The mice with a knock-out of ARID1A were conditionally depleted of ARID1A (10 mice) and simultaneously depleted of ARID1A and PTEN (12 mice), but they did not develop histological alterations. However, 40% of the mice with a simultaneous knock-out of ARID1A and PTEN developed poorly differentiated ovarian tumors, and 60% developed ovarian epithelium hyperplasia. The study concluded that ARID1A inactivation is not sufficient to initiate the carcinogenesis process, the alteration of the PI3K/PTEN/AKT pathway being a prerequisite in promoting tumor progression.

## 6. Circulating miRNAs as Biomarkers in Endometriosis

### 6.1. miRNAs—An Overview of Biogenesis and Function

MicroRNAs (miRNA) are single-stranded, non-coding RNAs of approximately 21–25 nucleotides long [47] and represent important epigenetic modulators of gene expression in a wide range of pathologies and physiological processes. At the endometrial level, miRNA is involved in the dynamic changes associated with the menstrual cycle and the pathophysiology of reproductive disorders, such as endometriosis and recurrent miscarriage. miRNAs are RNA fragments able to alter the function of distant cells and suppress the production of specific proteins to alter cell behavior [48].

The first miRNA was discovered over 20 years ago, and currently, there are over 2000 miRNAs described in humans [49]. It is believed that miRNAs collectively regulate one-third of the genes [49]. For decades, RNA was underestimated and thought to play a minor role in gene expression. However, in the late 1960s, a subset of RNA was found to control gene expression [50]. Since their discovery in 1993, a comprehensive database of miRNAs has been created and is constantly being updated, archiving all the published miRNA sequences and annotations with the latest release in October 2018, as miRBase 22.1 [51]. 

These non-coding RNAs are grouped into distinct classes based on their function and origin. These classes include microRNA (miRNA), small temporal RNA (stRNA), short interfering RNA (siRNA), short hairpin RNA (shRNA), small nuclear RNAs (snRNA), small nucleolar RNAs (snoRNA), transfer RNAs (tRNA), and ribosomal RNAs (rRNA) [52]. Following the completion of the Human Genome Project, it was found that there are almost 1000 genes in humans that encode miRNAs, accounting for approximately 3% of the human genome [53,54]. miRNAs are situated in the exons of non-coding genes, the introns of coding and non-coding genes, and the intragenic regions [55]. More than 60% of human protein-coding genes have conserved sites for miRNA [56]. Since they play a vital role in different physiological and pathological processes, such as cell-to-cell signaling, cellular division, differentiation, and death, various aberrations of miRNAs can cause multiple diseases, including cancer [57,58]. Almost 50% of human miRNA genes are present at genetic loci, which are involved in cancers [59].

MiRNAs are stored intracellularly, packed into extracellular vesicles, and released into circulation, where various chaperones carry them [60]. A study conducted by Vickers et al. pointed out that high-density lipoprotein (HDL) is also able to transport endogenous miRNAs and deliver them to recipient cells with functional targeting capabilities [61]. The circulating miRNAs are taken up by recipient cells, repressing the translation. 

The biogenesis of miRNAs is a complex process, and an individual miRNA can regulate more than a hundred mRNAs [62]. RNA Polymerase II (Pol II) transcribes miRNA genes, producing hundreds of primary miRNA transcripts, named pri-miRNAs. A pri-miRNA is composed of a double-stranded stem of approximately 33 base pairs, a terminal loop, and two flanking unstructured single-stranded segments [63]. After the first transcription, pri-miRNA is cropped into a 7-nucleotide hairpin-like precursor miRNA, named pre-miRNA [64,65,66]. 

Pri-miRNAs are capped at the 5’ end, polyadenylated, and finally spliced. A pri-miRNA can generate six miRNAs or more. The pri-miRNA is cleaved by a microprocessor complex formed by an RNase III enzyme, Drosha, and an RNA binding cofactor, Pasha, to form pre-miRNA. Drosha liberates the double-stranded stem from the remainder of the pri-miRNA by cleaving proximal and distal of the stem, and thus generates a pre-miRNA. The cleavage of pri-miRNA by microprocessor begins with DGCR8 [a double-stranded (ds) RNA binding protein] recognizing the ssRNA-dsRNA junction typical of a pri-miRNA [67,68,69,70]. 

Pre-miRNA is then exported from the nucleus into the cytoplasm by exportin-5 (Exp-5) through an energy-dependent process. Then, Dicer, which is an RNase III enzyme, cuts the loop at the 3’ and 5’ end, forming an miRNA duplex of 22 nucleotides with 3’ overhangs of two nucleotides. After the formation of this duplex, it will be unwound by enzymes to form two miRNAs. After a pre-miRNA is released from a pri-miRNA in the nucleus, it is exported by RanGTP and Exp-5 to the cytoplasm [69]. One of these miRNA will be destroyed, and one will bind to the Argonaute protein to be incorporated into the RNA-induced silencing complex (RISC) [71]. 

The principal mediators of miRNA function are Ago proteins. Binding of the miRNA to a target miRNA always occurs in the presence of an Ago protein. Base-pairing between nucleotides 2 and 8 of an miRNA and its cognate miRNA target is essential for binding miRNAs to their targets [72,73]. After the binding of miRNA to a target mRNA, the genes are silenced by mRNA cleavage or by translational repression. miRNAs bind to the 3′ untranslated region of specific mRNA targets, leading to translational repression or mRNA cleavage. They play an important role in the regulation of gene expression at the post-transcriptional level. Their importance resides in the intricate cooperative regulations that take place between mRNA and miRNA, since a single miRNA can control the expression of several mRNAs, and a single mRNA may be targeted by more than one miRNA [74]

### 6.2. miRNA Sequencing Methods

Numerous studies on human and animal models have highlighted the importance of miRNAs in both the developmental process as well as different pathologies, including endometriosis. miRNA profiling is an essential tool for the identification of differentially expressed miRNAs in normal and pathological processes and can be achieved by various techniques. Routine biopsies for miRNA profiling have not proven to be a practical option for early detection. As such, researchers are turning towards less invasive procedures, such as circulating miRNA from serum or plasma [75]. Since miRNAs are remarkably stable, disease-specific miRNA signatures have been investigated in various extracellular fluids [76,77]. The choice of a specific method depends on the body fluid, the number of miRNAs intended to be assessed, the available technology, and the cost [75]. 

miRNA profiling is a process that involves multiple steps, from the discovery phase, when miRNA expression profile is analyzed and compared between cases and controls, to the validation phase. There are several available methods that can be employed, including qRT-PCR, microarrays, sequence-specific hybridization in solution followed by miRNA molecules counting, and direct sequencing, each of them with their unique advantages and limitations [75]. Currently, the gold standard for miRNA profiling and quantification is qRT-PCR due to its high accuracy and sensitivity [78].

A novel miRNA quantification method has been developed using stem-loop RT followed by TaqMan PCR analysis. Stem-loop RT primers are better than conventional ones in terms of RT efficiency and specificity. Precise quantification is achieved routinely with as little as 25 pg of total RNA for most miRNAs. [79].

Similar to the TaqMan assay, the Qiagen SYBR green-based miScript PCR System enables the profiling of several hundred miRNAs from the same sample. The TaqMan assay is one of the most specific and sensitive methods for miRNA profiling and represents one of the most widely used [80]. 

Next-generation sequencing platforms have been introduced to sequence miRNAs. These novel platforms allow the sequence of millions of DNA fragments in parallel and include a wide range of categories, such as Solexa (Illumina®, San Diego, CA, USA), MiniSeq (Illumina®, San Diego, CA, USA), SOLid, MiSeq (Illumina®, San Diego, CA, USA), etc. Solexa sequencing provides a promising method for cancer-related miRNA profiling, and selectively expressed miRNAs could be used as potential serum-based biomarkers for ovarian cancer diagnosis [81]. The primary advantage of this technology is that it does not require knowledge of target miRNAs nor specific probes or primers and therefore does not limit studies to known miRNAs. [82]. The greatest advantage of the next-generation sequencing platforms approach is the potential for discovery of new small RNAs, such as ncRNAs. 

Thus, as future clinical investigations start filling the gaps of actual knowledge, the advantage of using not only extracellular miRNAs but perhaps other RNA species will significantly expand our understanding of the pathogenic mechanisms of endometriosis, ERONs, and possibly other diseases [75]. 

## 7. Results and Discussion

### 7.1. Differential miRNAs Expression in Endometriosis

miRNAs are considered paramount regulators of various cellular processes with high potential of becoming biomarkers of diagnostic and prognostic importance in endometriosis. Based on our analysis of the literature review, we determined that miRNA profiling was mainly achieved by microarray followed by qRT-PCR validation. Our study aimed to explore the role of circulating miRNAs as non-invasive biomarkers in endometriosis as well as the implication of these genes in ERONs diagnosis by finding the most specific miRNAs associated with endometriosis.

Since many miRNAs are tissue-specific, their dysregulation in peripheral blood points towards various pathologies, including endometriosis. miRNAs are biomarkers used not solely for diagnostic purposes but also for disease stratification [83]. miRNAs are very attractive as biomarkers due to their lower complexity, tissue specificity, lack of known post-translational modifications, and stability into blood, urine, or tissues [84]. As such, despite various limitations of these molecules, they have given a whole new dimension to the field of biomarkers.

One of the most studied miRNAs associated with endometriosis is the miR-200 family, consisting of miR-200a, miR-200b, and miR-141. These miRNAs are dysregulated in both blood and tissues of endometriotic patients. Ohlsson et al. [85] conducted a study to highlight the association of these three members of the miR-200 family with endometriosis. Their results revealed that the combination of these three circulating miRNAs had a sensitivity and a specificity of 84.4% and 66.7%, respectively. Based on the expression of the miR-200 family in the tissues of endometriotic women, Ohlsson [85] and Hawkins [86] pointed out in their studies that these miRNAs were downregulated in ectopic endometrium compared to the eutopic endometrium, and that miR-200b exhibited a decreased expression in endometriomas compared to normal eutopic endometrium.

Ectopic endometrium has many characteristics of malignant cells, such as invasiveness, high proliferation rate, and metastasis. It has been proven that a significant down-regulation in the miR-200 family induces an epithelial-mesenchymal transition characteristic of endometriosis [87]. As such, the down-regulation of these miRNAs can be associated with endometriosis as part of the pathophysiology of this disease. 

Another circulating miRNA considered to be a leading biomarker for endometriosis is miR-20a. Our analysis revealed that it was down-regulated in several studies and thus seems to play an important role in the aetiopathogenesis of endometriosis [88,89]. The sensitivity and the specificity of these biomarkers were increased. MiR-20a targets TGF-β and Il-8, and its down-regulation leads to increased concentrations of these cytokines. Considering the role of TGF-β and Il-8 in promoting inflammation and tissular repair, the down-regulation of miR-20a, which targets these factors, may explain the growth of endometriotic lesions. miR-20a was also found up-regulated in the ovarian tissue of patients with ovarian endometriosis and promoted neovascularization [90]. It is worthwhile mentioning that miR-20a is not specific for endometriosis, as it has been found dysregulated in various cancers, including ovarian cancer. Furthermore, it cannot distinguish between primary ovarian cancer and ERONs. miR-143 has been found to be up-regulated in the serum of affected women. It is associated with cell invasion and intensive migration, which are characteristic of endometriosis. Increased expression of miR-143 will repress the transcription of its target, FNDC3B (fibronectin type III domain containing 3B), and promote cell invasion and migration [91]. 

Wang et al. [92] revealed in their study that miR-199a is another circulating miRNA with sensitivity and specificity of 78.33% and 76% for endometriosis. This marker was significantly down-regulated in both ectopic endometrium and ovarian endometriomas compared to eutopic endometrium [93]. miR-99a targets Nf-kB and Il-8, which enhance the invasiveness of endometrial cells. Thus, the down-regulation of miR-199a will increase the expression of Nf-kB and Il-8, leading to increased invasive capability [94]. In contrast, other studies refer to an up-regulation of miR-199a in women with endometriosis. These miRNAs also target CLIC4 and VCL and produce a deep infiltration of endometrial cells [92]. 

Similarly, miR-145 expression in endometriosis is also controversial. While some studies have observed the down-regulation of miR-145 [92], others imply that miR-145 is up-regulated in this gynecological disease [95]. Furthermore, miR-145 was found to be down-regulated in patients with stage 1 and 2 endometriosis, but not with advanced stages [51]. 

In Table 1, we summarize the studies illustrating the sensitivity and the specificity of various dysregulated miRNAs in endometriosis. 

### 7.2. miRNAs Dysregulations in Endometriosis 

The analysis of circulating miRNAs for endometriosis has increased the interest regarding their involvement in the large biomarker panel of endometriosis. Therefore, many studies aimed at highlighting the usefulness of this non-invasive biomarker in the early diagnosis of endometriosis. miRNAs are considered potent regulators of gene expression in the pathogenesis of endometriosis, since cell survival, the remodeling of the matrix, proliferation, and angiogenesis are essential in the pathophysiology of this disease and are potentially regulated by miRNAs [100].

The most used techniques in miRNA expression assessment are next-generation sequencing and microarrays [101]. The first step in defining a pathological miRNA expression is to evaluate its pattern in the pathological tissue or different fluids of the body, including urine, serum, plasma, or cerebrospinal fluid [102]. 

A preclinical study performed by Seifer et al. [103] used miRNA array to investigate miRNA abundance in the serum of mice with experimental endometriosis. Their results indicated that let-7a-5p was decreased in the serum samples of the mice. Serum let-7 family mRNA revealed similar dysregulation in endometriosis in both humans and mice with c-5p and e-5p downregulated. This study suggested that miRNAs may be involved in endometriosis and may be used as biomarkers, but further investigation to determine the functional role of let-7 miRNA is necessary. 

Several clinical studies have reported differences between miRNA expression in the eutopic endometrium from women with and without endometriosis. Cho et al. [96] evaluated in their study whether miRNA is detectable in the circulation of patients with endometriosis. They used serum samples collected from patients undergoing laparoscopy for endometriosis and compared the results with samples collected from healthy controls. They concluded that the circulating levels of let-7b and miR-135a were significantly decreased in affected women compared with controls, while let-7d and 7f showed a down-regulating trend. Furthermore, the expression of let-7b was strongly correlated with higher serum CA-125 levels in endometriosis women, and let-7b, 7c, 7d, and 7e were significantly lower during the proliferative phase of the menstrual cycle compared to controls. 

In a study addressing the serum miRNA expression profile in the endometrium of women with endometriosis compared to a control, Wang et al. [92] analyzed 765 serum samples using a TaqMan microRNA array. The expression of miR-199a and miR-122 revealed an ascending trend in endometriosis compared with controls, while miR-145*, miR-141*, miR-542-3p, and miR-9* had a decreased expression in endometriosis. The authors also highlighted that miR-199a was well correlated with pelvic adhesion and lesion distribution of endometriosis. Therefore, based on these results, miRNAs miR-199a, miR-122, miR-145*, and miR-542-3p could potentially be included as noninvasive biomarkers for this pathology.

Laudanski et al. [104] enrolled 21 women with ovarian endometriosis and 25 healthy women and analyzed the miRNA expression in their serum samples by PCR arrays. The researchers pointed out that miR-483-5p and miR-629-3p were downregulated in the eutopic endometrium of patients compared to controls. In conclusion, the down-regulation of these genes could be a determinant factor of endometrial tissue overgrowth. 

Rekker et al. [98] performed an investigation on 61 patients and 65 control women. They studied the serum expression of the miR-200 family (miR-200a-3p, miR-200b-3p, and miR-141-3p) in the included cohorts. They concluded that the expression of all these members was downregulated in patients compared to controls, and that miR-200a-3p and miR-141-3p had the highest potential in being used as noninvasive biomarkers for endometriosis. Another aspect of miRNA panel analysis consisted of the circadian variations assessment. The study revealed lower evening levels, an important observation that should be further investigated in the analysis of circulating miRNAs expression. 

In 2013, Jia et al. [88] conducted a 46 cases study with 23 women with endometriosis and 23 endometriosis-free controls. The miRNA expression pattern was analyzed by qRT-PCR, and the authors concluded that miR-17-5p, miR-20a, and miR-22 were significantly downregulated in affected women compared to healthy subjects. 

Suryawanshi et al. [97] used global profiling of more than 1000 miRNAs based on qRT-PCR in a 20-women screening cohort. In this screening, 23 miRNAs differentially expressed between healthy controls and endometriosis-affected subjects were identified, contributing to the idea that distinct plasma miRNA expression patterns may be considered specific noninvasive biomarkers for endometriosis. 

Grechukhina et al. [105] reported a polymorphism in the LCS6 let-7 miRNA binding site of the KRAS 30-UTR that was detected in almost 31% of all endometriosis cases and was significantly higher than controls. In women harboring this KRAS mutation, the altered miRNA led to uncontrolled proliferation of endometrial stromal cells, invasion, and ultimately endometriosis development.

Literature review reveals only one study that investigated the expression of miRNAs in a nonhuman primate model. Cosar et al. [106] evaluated the levels of circulating miRNAs in 16 baboons with induced endometriosis following a simvastatin treatment for 90 days. The primates were divided into two groups; one group received simvastatin, and the second group received vehicle only. The endometriosis lesions were evaluated after 90 days by laparoscopy. The authors observed decreased levels of miR-150-5p and miR-451a in the treated group, as well as increased levels of miR-3613-5p compared to the untreated group. These results were similar to those obtained in previous human studies. As such, circulating miRNAs suggest a high potential of being used as biomarkers not only for diagnosis purposes but also for disease progression and therapy response. The lack of studies on humans regarding the potential of miRNAs as prognostic biomarkers in endometriosis highlights the necessity for further research in this direction. 

A review of the clinical studies that analyzed miRNAs expression in endometriosis is presented in Table 2.

### 7.3. Circulating miRNAs as Biomarkers in ERONs and Ovarian Cancer

The purpose of miRNAs is to express various genes by repressing the translation of or causing the degradation of multiple-target mRNAs [95]. In cancer, miRNAs play a pivotal role as regulatory molecules, acting as oncogenes (oncomiRs) or tumor suppressors [108]. Based on the tissue-specific dysregulation of miRNA expression profiles in cancer, the potential usefulness of these molecules has been greatly explored over the past decades. With an accurate detection of miRNA quickly realized in small biopsy specimens or extracellular fluids, researchers revealed the potential of miRNAs from tumoral tissues as predictors of tissular origin. 

The assays of miRNA in blood samples have been developed as novel, minimally invasive biomarkers for the detection and the risk assessment of ovarian cancer, and qRT-PCR is the most popular method used for gene expression quantification of miRNA [107]. Mitchell et al. pointed out that miRNA from cancer tissues are protected from RNase activity, and these molecules might be used as circulating biomarkers for various types of cancer [109]. 

Several studies concluded that serum or plasma miRNAs can discriminate women with ovarian cancer from healthy patients, and that cell-free miRNAs circulating in body fluids such as blood, urine, serum, or plasma may reflect not only the existence of a tumoral mass but also tumor histology, stage, or prognosis. We identified only one study that aimed to determine if plasma miRNAs could be used to differentiate patients with ovarian cancer from non-affected women. Suryawanshi et al. [97] described three distinct miRNAs with strong differential expression between healthy people and patients with ERONs. Furthermore, the majority of miRNAs expressed in human ERONs were mirrored in a mouse model. The authors also measured the expression of miR-15b, 16, 21, 191, and 195 in a mice model, since only these five miRNAs have mouse orthologs. Their results revealed that four of the five miRNAs could also delineate the EAOC (Endometriosis-associated ovarian carcinoma) mice from healthy controls, suggesting potential EAOC-specific pathogenesis leading to the dysregulation of miR-15b, 16, 21, and 195. Moreover, Suryawanshi and coworkers paired tumor and plasma samples from tumor-bearing KrasG12D/þ/Ptenloxp/loxp mice to support the hypothesis that miRNAs in the plasma do not reflect miRNAs expression in corresponding diseased tissues. They proved that no significant correlation was detected in the paired samples from all five mice, mirroring the findings in humans.

A high number of studies have investigated plasma and serum miRNA expression patterns in ovarian cancer compared to ERONs. After analyzing miRNA profiles from 24 patients with ovarian cancer, Hausler et al. [110] concluded that ovarian cancer-associated miRNA profiles are not yet sensitive and specific enough, but if combined with other markers, they have the potential of being used for ovarian cancer screening. 

Kinose et al. [111] pointed out that several serum miRNA profiles are unique for ovarian cancer and may be used as diagnostic and prognostic biomarkers. miRNAs such as the miRNA-200 family, the miR 199/214 cluster, and the let-7 paralogs have been found dysregulated in chemoresistant ovarian tumors. The miR-205 expression was significantly up-regulated and the let-7f was down-regulated in plasma of EOC women when compared with healthy individuals, according to Zheng et al. [112]. 

miRNA could also distinguish women with high-grade serous ovarian cancer from healthy women. Kan et al. [113] assessed serum miRNA levels from 28 ovarian cancer patients and 28 controls using qRT-PCR following preamplification. miR-182, miR-200a, miR-200b, and miR-200c were up-regulated in high-grade serous ovarian cancer subjects compared to controls. Furthermore, up-regulated miR-103 was the best predictive classifier of ovarian cancer. The researchers concluded that determination of miRNAs levels might improve the diagnosis of ovarian cancer. 

Guo et at. [114] prospectively investigated the effectiveness of miRNA-92 as a biomarker for EOC. RT-PCR measured miRNA-92 levels in serum samples from 50 patients and 50 controls, which revealed that miRNA-92 was significantly higher in EOC. Furthermore, the study reported a significant correlation between miRNA-92 and the clinical stage of ovarian cancer. 

Clear cell carcinoma of the ovary is one of the most common histological types of ERONs. With the aim of identifying the usefulness of miRNAs in monitoring CCC progression and their potential to serve as biomarkers for this disease, Chao et al. [115] analyzed the serum of patients with CCC and performed 270 miRNA profiles by RT-PCR. Four miRNAs, namely hsa-miR-130a, hsa-miR-138, hsa-miR-187, and hsa-miR-202, were elevated in the serum of preoperative patients. Their results pointed out that miRNA may be a useful tool in detecting CCC as well as the recurrences of CCC, but further studies are necessary.

Resnick et al. [116] used serum samples from EOC patients and extracted miRNAs using RT-PCR. The results were compared to normal serum samples using the TaqMan Array Human MicroRNA panel. miRNAs-21, 92, 93, and 29a were significantly over-expressed in the serum of cancer patients, while miRNAs-155, 127, and 99b were significantly under-expressed in the same samples compared to healthy subjects. Therefore, it can be inferred that these miRNAs might be used as biomarkers in ovarian cancer with further research.

The down-regulation of miRNAs expression in ovarian cancer samples compared to control samples was found in miR-106a, miR126, miR-146a, miR-150, miR-16, miR-17, miR-19b, miR-20a, miR-223, miR-24, and miR-92a. According to Shapira et al. [117], the down-regulated expression of miRNA was also observed in miR-106b, miR-191, miR-193a-5p, miR-30b, miR-30a- 5p, miR-30c, miR-320, and miR-328. 

The role of miRNAs as biomarkers in ovarian cancer has been highlighted in a wide range of studies. Alternatively, with respect to the ERONs, there is still a lack of information, and further studies are necessary to establish the effectiveness of these molecules in detecting endometriosis related-ovarian cancer. 

## 8. Conclusions

Despite being a benign gynecological disease, endometriosis is a debilitating pathology that profoundly impairs the quality of life in terms of infertility, chronic pain, and vaginal bleeding. The gold standard for endometriosis diagnosis remains as laparoscopy followed by histological confirmation, but novel non-invasive potential biomarkers of endometriosis offer promising perspectives and should be further investigated. 

Endometriosis has a malignancy potential. Various stimuli may disturb the normal evolution of this pathology and convert it to endometriosis-related ovarian cancer. Although research results are still equivocal, circulating mRNAs have emerged as attractive molecules to be considered as biomarkers for endometriosis and ERONs, and further research should be conducted to validate miRNA as a diagnostic tool. 

Furthermore, miRNA expression profiles may be affected by pharmacological treatments and clinical conditions. While only one study demonstrated the efficacy of miRNAs as biomarkers of ERONs, there is a growing body of proof regarding the efficacy of these molecules as biomarkers in ovarian cancer. 

Although multiple studies were conducted on endometriosis, no single miRNA was considered as a sole biomarker for this pathology. However, since the prognostic value of biomarkers is generally enhanced if more are assessed at the same time, a panel of miRNAs could be a better indicator of the disease. The seemingly conflicting results of different studies highlight the need for further extended research to prove if miRNAs could become viable biomarkers for endometriosis and ERONs. 

## Figures and Tables

**Table 1:** Reported sensitivity and specificity of dysregulated microRNAs (miRNAs) in endometriosis.

Author, Reference	miRNA	Sensitivity (%)	Specificity (%)
Jia, 2013 [88]	miR-20a	60	90
	miR-22	90	90
	miR-17-5p	60	80
Cosar, 2016 [95]	mi-125b-5p	100	96
Cho, 2015 [96]	let-7d	83.3	100
Wang, 2013 [92]	miR-122	80	76
	miR-141-5p	71.69	96
	miR-145	70	96
	miR-199a	78.33	76
Suryavanshi, 2013 [97]	miR-16+miR-191+miR-195	88	60
Rekker, 2015 [98]	miR-141	71.9	70.8
	miR-200a	90.6	62.5
	miR-200b	90.6	70.8
Nisenblat, 2019 [99]	miR-155+miR574-3p+miR139-3p	83	51

**Table 2:** Studies reporting miRNAs expression in endometriosis.

Author, Year, Reference	Biofluid	Cases	mRNAs Sequencing Method	Results
Wang, 2016 [89]	Serum	30 cases of stage I-II endometriosis and 20 controls	Deep sequencing	Up-regulated: miR-185-5p, miR-242-5p, miR-296-5p, miR-3127-5p, miR-424-3p, miR-4645-3p, miR-502-3p, miR-542-3p, miR-550a-3p, miR-636
Hsu, 2014 [93]	Serum	40 cases of endometriosis and 25 controls	Array profiling	Down-regulated: mir-199a-5p
Cosar, 2016 [95]	Serum	24 cases of stage III-IV endometriosis and 24 controls	Array profiling	Down-regulated: miR-3613-5p, miR-6755-3p Up-regulated: miR-500a-3p, miR-451a, miR-18a-5p, miR-342-3p, miR-125b-5p.
Burney, 2009 [107]	Serum	4 cases of endometriosis and 3 controls	Array profiling	Down-regulated: miR-34c-3p, miR-9*, miR-34b*, miR-34c-5p, miR-9
Suryawanshi, 2013 [97]	Plasma	33 cases of and 20 controls	Array profiling	Up-regulated: miR-16, miR-191, miR-195
Cho, 2015 [96]	Serum	24 cases of stage III-IV endometriosis and 24 controls	Targeted	Down-regulated: let7b, miR-125a

## Abbreviations

*ARID1A*	AT-rich interactive domain-containing protein 1A
*BRAF*	V-raf murine sarcoma viral oncogene homolog B1
*CA-125*	Cancer-associated carbohydrate antigen 125
*CCC*	Clear cell carcinoma
*CLIC4*	Chloride intracellular channel 4
*CTNNB1*	Gene encoding catenin beta-1
*DGCR8*	Double stranded RNA binding protein
*DNA*	Deoxyribonucleic acid
*EAOC*	Endometriosis-associated ovarian carcinoma
*EnOC*	Endometrioid ovarian carcinoma
*EOC*	Epithelial ovarian cancer
*ER*	Estrogen-receptors
*ERONs*	Endometriosis-related ovarian neoplasms
*Exp-5*	Exportin 5
*FGF-2*	Fibroblast growth factor-2
*HDL*	High density lipoprotein
*IL-6*	Interleukin 6
*IL-8*	Interleukin 8
*KRAS*	V-Ki-ras2 Kirsten rat sarcoma viral oncogene homolog
*let-7*	A micro-RNA precursor
*miR*	Micro- RNA precursor family
*miRNA*	Micro Ribonucleic acid
*MMPs*	Matri metalloproteinases
*p53*	Protein 53
*PI3K*	Phosphatidylinositol 3-kinase
*PIK3CA*	Phosphatidylinositol-4,5-bisphosphate 3-kinase catalytic subunit alpha
*Pol II*	RNA Polymerase II
*PPP2R1A*	Protein phosphatase two scaffold subunit Aalpha
*PTEN*	Phosphatase and tensin homolog
*qRT-PCR*	Quantitative Reverse transcription polymerase chain reaction
*RCBTB1*	Regulator of chromosome condensation and BTB domain-containing protein-1
*RISC*	RNA induced silencing complex
*rRNA*	Ribosomal RNA
*RT-PCR*	Reverse transcription polymerase chain reaction
*shRNA*	Short hairpin RNA
*siRNA*	Short interfering RNA
*SMAD3*	Mothers against decapentaplegic homolog 3
*snRNA*	Small nuclear RNA
*snoRNA*	Small nucleolar RNA
*SMARCB1*	A gene that encodes SWI/SNF related matrix-associated actin-dependent regulator of chromatin subfamily B member 1
*stRNA*	Small temporal RNA
*SWI/SNF*	Switch/Sucrose Non-Fermentable
*TGF-β*	Transforming growth factor beta
*TNF-α*	Tumor necrosis factor alpha
*TP53*	Tumor protein p53
*tRNA*	Transfer RNA
*WWOX*	Ww domain-containing oxidoreductase
